# Detection and Separation of Inorganic Cations in Natural, Potable, and Wastewater Samples Using Capillary Zone Electrophoresis with Indirect UV Detection

**DOI:** 10.4236/ajac.2017.81007

**Published:** 2017-01

**Authors:** Lara Varden, Fadi Bou-Abdallah

**Affiliations:** Department of Chemistry, State University of New York (SUNY) at Potsdam, Potsdam, NY, USA

**Keywords:** Capillary Zone Electrophoresis, Indirect UV Detection, Inorganic Cations, Northern New York Raquette River, Adirondack Watershed

## Abstract

Capillary zone electrophoresis (CZE) is a sensitive and rapid technique for determining traces of inorganic cations in water samples. CZE with indirect UV-diode array detection (CZE-DAD) was utilized to identify several inorganic cations in natural, potable, and wastewater samples. A pH 4.35 background electrolyte system was employed and consisted of 15 mM imidazole, 8 mM malonic acid, 2 mM 18-crown-6 ether as complexing agents, 10% v/v methanol as an organic modifier with indirect absorbance reference at 214 nm. The CZE method involved electromigration injection at 5 kV for 5 s, a separation voltage of 20 kV at 25°C, and a detection wavelength of 280 nm. Six main cations (ammonium ${\text{NH}}_{4}^{+}$, potassium K^+^, calcium Ca^2+^, sodium Na^+^, magnesium Mg^2+^, and lead Pb^2+^) were tested, and all but lead, were detected in the water samples at concentrations between 0.03 and 755 ppm with a detection limit ranging between 0.023 and 0.084 ppm. The successful evaluation of the proposed methodology allowed us to reliably detect and separate six metal ions in different water samples without any pretreatment. All water samples were collected from Northern New York towns and the Raquette River water system, the third longest river in New York State and the largest watershed of the central and western Adirondacks.

## 1. Introduction

In 1937, Tiselius introduced electrophoresis as a separation technique for which he received a Nobel Prize [1]. In 1981, Jorgenson and Lukas modernized the technique by utilizing fused silica capillaries while also clarifying the theory of the relationship between separation quality and operational parameters. They demonstrated capillary electrophoresis (CE) separation as an analytical technique based on the differences in solute velocity in an electric field [2]. Since its inception, CE has become a well-established analytical technique used in laboratories worldwide. Compared to other separation techniques, the CE offers short analysis times, highly efficient separation, and minimal consumption of solvents, reagents and samples. CE has shown to be quite successful and efficient in the analysis of small molecules and has even produced higher separation efficiency compared to HPLC (High Performance Liquid Chromatography) [3], ASS (atomic absorption spectrometry, IC (ion chromatography), and ICP-MS (inductively coupled plasma with mass spectrometry) [4] in testing of water samples while consuming lower sample volumes [5]. In capillary zone electrophoresis (CZE), one of many electrophoretic techniques of CE, the molecules are separated based on their size to charge ratio and there exist numerous examples in the literature [6]-[12]. When implementing commercial CE instruments for the separation and analysis of non-absorbing ions, indirect-UV methods are most advantageous. Notwithstanding that the indirect technique is not as sensitive as the direct technique, the separation medium must provide quality resolution of the ion zones, precise sample zones, and a running buffer with high direct-UV absorbance in order to detect low ions concentration in actual samples [13].

Nine essential inorganic ions (Na^+^, K^+^, Ca^2+^, Mg^2+^, H^+^, Cl^−^, ${\text{HCO}}_{3}^{-},\;{\text{PO}}_{4}^{3-}$, and OH^−^) are naturally present in water with a significant role in supporting and maintaining health and life [14]. Unfortunately, over the last few decades, water supplies used for drinking, washing, agriculture, fishing and even recreation have become more and more contaminated on a global scale. The quality of water is regulated in the United States via the Clean Water Act (CWA) of 1972 [15], which primarily governs water pollution of surface waters, and the Safe Drinking Water Act (SDWA) of 1974 that deals mainly with sources of drinking water and groundwater contamination. These water Acts are instituted federally by the United States Environmental Protection Agency (US EPA). The SDWA only regulates 91 contaminants, yet the United States uses in excess of 80,000 chemicals, according to US EPA estimates. The New York State Department of Environmental Conservation (NYSDEC) water quality standards program is a state program with federal oversight by the US EPA, which pre-exists the federal Clean Water Act and protects surface waters as well as ground waters. Table 1 shows a list of cations of interest to this study listing US EPA’s and NYSDEC’s [16] regulation standards, source or cause, and health significance.

In this study, the main objective was to test and optimize CE methods to detect and quantify certain cations present in natural water, drinking water, and wastewater samples from the Raquette River water system and other local communities. Water samples were obtained at numerous sites in the Northern New York area, primarily of the Raquette River water system, which is the third longest river in New York State and the largest watershed of the central and western Adirondacks. The river system begins at Blue Mountain Lake, flows south to Raquette Lake, turns northeast to Long Lake, heads north to Tupper Lake through the Adirondack Park, continuing north where it finally empties into the St. Lawrence River, northeast of Massena and bordering Canada. The Raquette River is one of the most popular, recreationally traveled and fished rivers of the Adirondack Park. Historically, every community along the River drew their drinking water from this river system; but due to changes in water quality of this watershed, only a handful of communities still draw their drinking water out of this source. The rest have been forced by the New York State Department of Health (NYSDOH) regulations on water quality to obtain their drinking water from deep-water wells. The village of Potsdam in Upstate New York resides on the banks of the Raquette River and is the largest user of drinking water drawn off the River in addition to two local universities, State University of New York at Potsdam and Clarkson University.

## 2. Materials and Methods

### 2.1. Chemicals

Lead chloride and glacial acetic acid were purchased from Fisher Scientific (Fairlawn, NJ, USA), imidazole and malonic acid were obtained from Aldrich (Milwaukee, WI, USA), 18-crown-6 ether was acquired from Sigma-Aldrich (St. Louis, MO, USA), and methanol was attained from Pharmco-Aaper (Brookfield, CT, USA). All reagents were of analytical grade. Ultra pure CE water obtained from Agilent Technologies (Santa Clara, CA, USA).

### 2.2. Standards and BGE

Agilent’s Cation Test Mixture stock (100 ppm each of ammonium, potassium, calcium, sodium, magnesium) was diluted to 25 ppm with DI water and 250 ppm lead from 1000 ppm stock of lead (chloride salt) dissolved in DI water obtained from EMD Millipore Elix10 Water Purification System (Darmstadt, Ger-many). The standard mixture was filtered using a 0.22 μm PES syringe filter (Argos Technologies, Elgin, IL, USA). The running buffer or BGE (background electrolyte) consisted of 15 mM imidazole, 8 mM malonic acid, 2 mM 18-crown-6 ether, 10% v/v MeOH with pH adjusted to 4.35 using 50% v/v acetic acid. All chemicals were dissolved in DI water. Buffer was filtered using 0.22 μm PES membrane syringe filter and degassed for 10 min using TA Instruments degassing station (New Castle, DE, USA) in 2 mL glass CE vials (Agilent Technologies) prior to runs.

### 2.3. Instrumentation

The capillary electrophoresis system employed in this study is the Agilent G7100 with a UV-DAD (diode array detector) interfaced with ChemStation software for data acquisition (Agilent Technologies, Santa Clara, CA, USA). The separation capillary used was a standard bare fused silica by Agilent Technologies measuring 64.5 cm total length, 56 cm effective length, and an internal diameter (i.d.) of 75 μm.

### 2.4. Procedures

Prior to first use and daily conditioning of capillary included flushing with 0.1 M NaOH for 10 min, ultra-pure water for 10 min, and running buffer/BGE for 15 min. The capillary was conditioned between runs with 5 min of BGE. Conditioning of the capillary at the end of the day included 15 min ultra-pure water flush and 3 min air flush. The separation was completed with a voltage at 20 kV (positive polarity) and the capillary cassette was kept at 25°C for all experiments. Sample injection was performed under electromigration injection at 5 kV for 5 s. Detection was at UV signal of 280 nm (20 nm bandwidth) with an indirect reference signal of 214 nm (10 nm bandwidth), and a runtime of 12 min. Each sample was run at least three times to ensure reproducibility and the results in Table 2 are average values from multiple experiments with less than 5% uncertainty.

### 2.5. Samples

**Figure 1:**
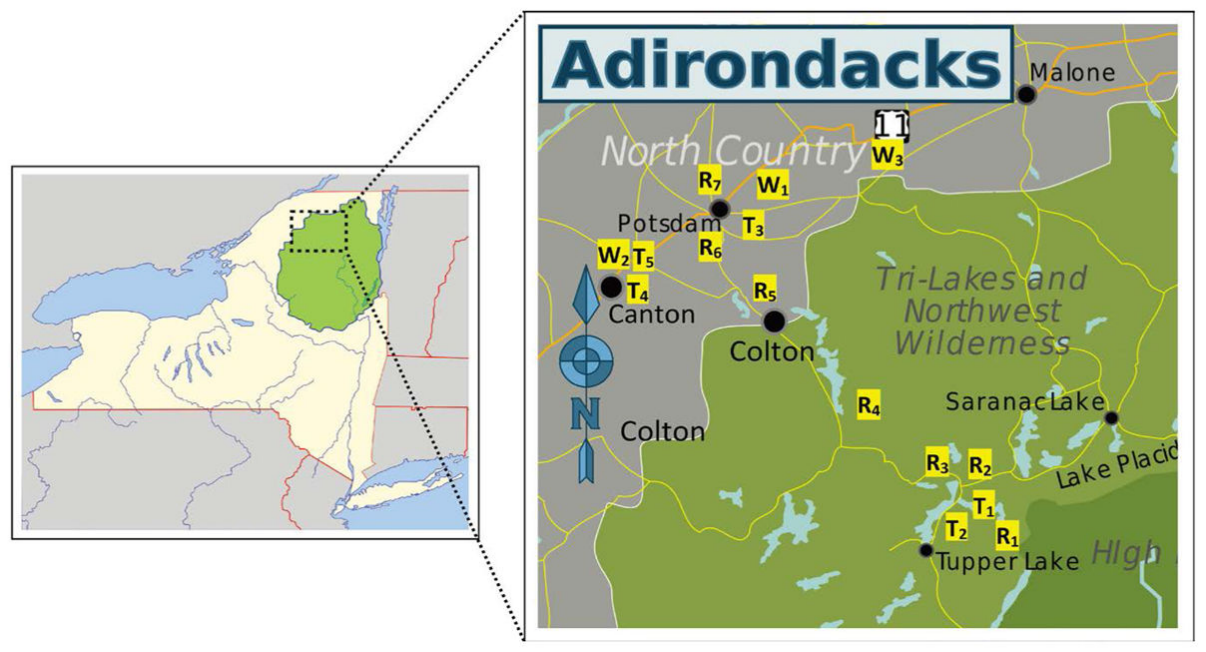
*Left map*: Map of New York State and surrounding states in the US, southeastern Canada with shadow/grey box representing the collection site area. *Right map*: Sample site map of Raquette River water system sites, including pre- and post-treated waste water from Tupper Lake and Potsdam, (R), tap water sites (T), and well water sites (W) in northern New York State, USA. The left image is credited to Jackaranga and Daniel Case under the license “Creative Commons CC BY-SA 4.0”. https://commons.wikimedia.org/wiki/File:Adirondack_Park_map_with_Blue_Line.svg The right image is credited to Peter Fitzgerald, Jackaranga, Algorerhythms, and Daniel Case under the license “Creative Commons CC BY-SA 4.0”. https://commons.wikimedia.org/wiki/File:Adirondacks_regions_map.svg

All water samples (natural river waters, potable tap and well waters, and waste-waters, pre- and post-treatment) were collected in BPA-free plastic gallon water containers and filtered through a 0.22 μm PES syringe filter by Agros. No further manipulation of water samples (*i.e*. dilution, concentration, pH adjustment, complexation, etc.) was performed so as to mimic natural water conditions during testing. Water samples were obtained from water sources feeding areas of interest, mainly Raquette River system from Tupper Lake (TL), NY, to Potsdam, NY, as well as pre- and post-wastewater treatment (WWT) from the two villages. Tap and well waters from other local areas including Canton and Winthrop, NY, were also tested. Figure 1 depicts Northern New York map of sample site locations.

## 3. Results and Discussion

### 3.1. Comparison and Optimization of the CE Method

**Figure 2:**
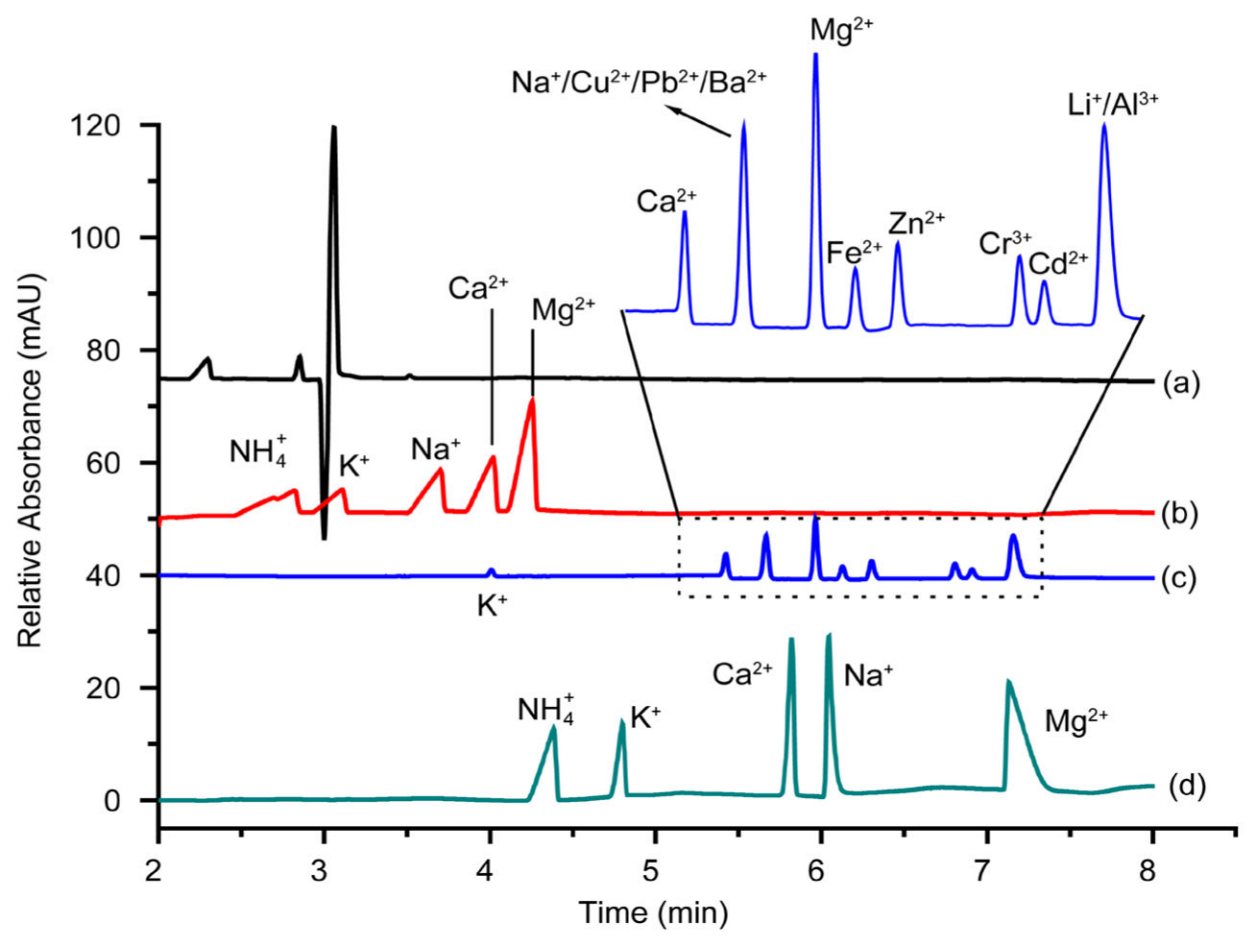
Comparison of electropherograms showing separations of standard cation mixtures using different methods and/or BGEs. The standard cation mixtures were prepared at a concentration of 100 ppm for each cation (ammonium, potassium, calcium, sodium and magnesium). (a) BGE: 5 mM imidazole, pH 5.1; Separation conditions: 30 kV, 25°C, hydrodynamic injection of 35 mbar for 5 s, indirect UV detection at 215 nm; Capillary: bare fused silica, 50 μm i.d., 56 cm length. (b) BGE: Agilent Cation Kit proprietary electrolytes; Separation conditions were the same as in (a); Capillary: extended light path (bubble factor 3, optical path of 150 μm) bare fused silica, 50 μm i.d., 56 cm length. (c) BGE: 15 mM Imidazole, pH 3.7 (using 17 M glacial acetic acid); Separation conditions: 20 kV, 25°C, hydrodynamic injection of 35 mbar for 5 s, indirect UV detection at 214 nm; Capillary: same as in (b); (d) BGE: 15 mM Imidazole, 8 mM malonic acid, 2 mM 18 crown-6, and 10% v/v methanol, pH 4.35 (using 50% v/v acetic acid); Separation conditions: 20 kV, 25°C, electromigration injection at 5 kV for 5 s, indirect UV detection at 214 nm; Capillary: bare fused-silica, 75 μm i.d., 56 cm length.

Figure 2 displays the electropherograms of standard cation mixtures being separated using varying methods, BGE, and capillaries. The standard cation mixtures represented by electropherograms (a), (b) and (d) of Figure 2 incorporated 5 cations of 100 ppm each (ammonium, potassium, calcium, sodium and magnesium). The cation standard mixture used in the method illustrated by electro-pherogram Figure 2(c) included 13 cations (K^+^, Ca^2+^, Na^+^, Cu^2+^, Pb^2+^, Ba^2+^, Mg^2+^, Fe^2+^, Zn^2+^, Cr^3+^, Cd^2+^, Li^+^, and Al^3+^) of 3 ppm each with only 9 signals showing up. Six of these cations were unsuccessfully separated. Cations Na^+^, Cu^2+^, Pb^2+^ and Ba^2+^ elute at the same time with sodium most likely masking the other three ions. Cations Li^+^ and Al^3+^ also elute at the same time.

Sensitivity and selectivity are affected by the background electrolyte (BGE) composition as well as concentration [12]. Separation efficiency is proportional to the concentration of the running buffer [17]. Therefore, the higher the BGE concentration the better the separation efficiency, although too high of BGE concentration could cause large joule heat in the capillary during the run [12]. The running buffer contained imidazole, which acts as background absorption for indirect UV detection. With migration velocities of cations being relatively close together, it can be hard to separate them directly by CE; thus, a weak complexing agent can be used to improve separation efficiency. Since heavy metal ions tend to precipitate out of solution in basic environments, a more acidic solution is preferable for the separation of metal cations.

The results presented in Figure 2 are those of several cation mixtures using different methods and/or BGEs. The detailed experimental conditions for each method are indicated in the figure caption. The electropherogram of Figure 2(a) did not provide decent detection, separation, or selectivity of the cations which may be due to a number of factors including a low running buffer concentration, the absence of a complexing agent, and a high pH. The separation of the cations as observed in Figure 2(b) electropherogram was decent, but the peaks were broad and non-symmetrical. The electropherogram shown in Figure 2(c) exhibited a good separation of most of the cations notwithstanding the aforementioned issues with some cation peaks overlapping. Out of the four tested methods, the best separation of cations, baseline stability, and sensitivity was achieved with a BGE that consisted of 15 mM imidazole, 8 mM malonic acid, 2 mM 18 crown-6, 10% v/v methanol, pH 4.35 (using 50% v/v acetic acid) with separation conditions of 20 kV, 25°C, an electromigration injection of 5 kV for 5 s, a bare fused-silica capillary of 75 μm i.d. and 56 cm effective length, and an indirect UV detection of 214 nm (Figure 2(d)). This method was therefore adopted in this study.

### 3.2. Cation Analysis of Standard Mixture

**Figure 3:**
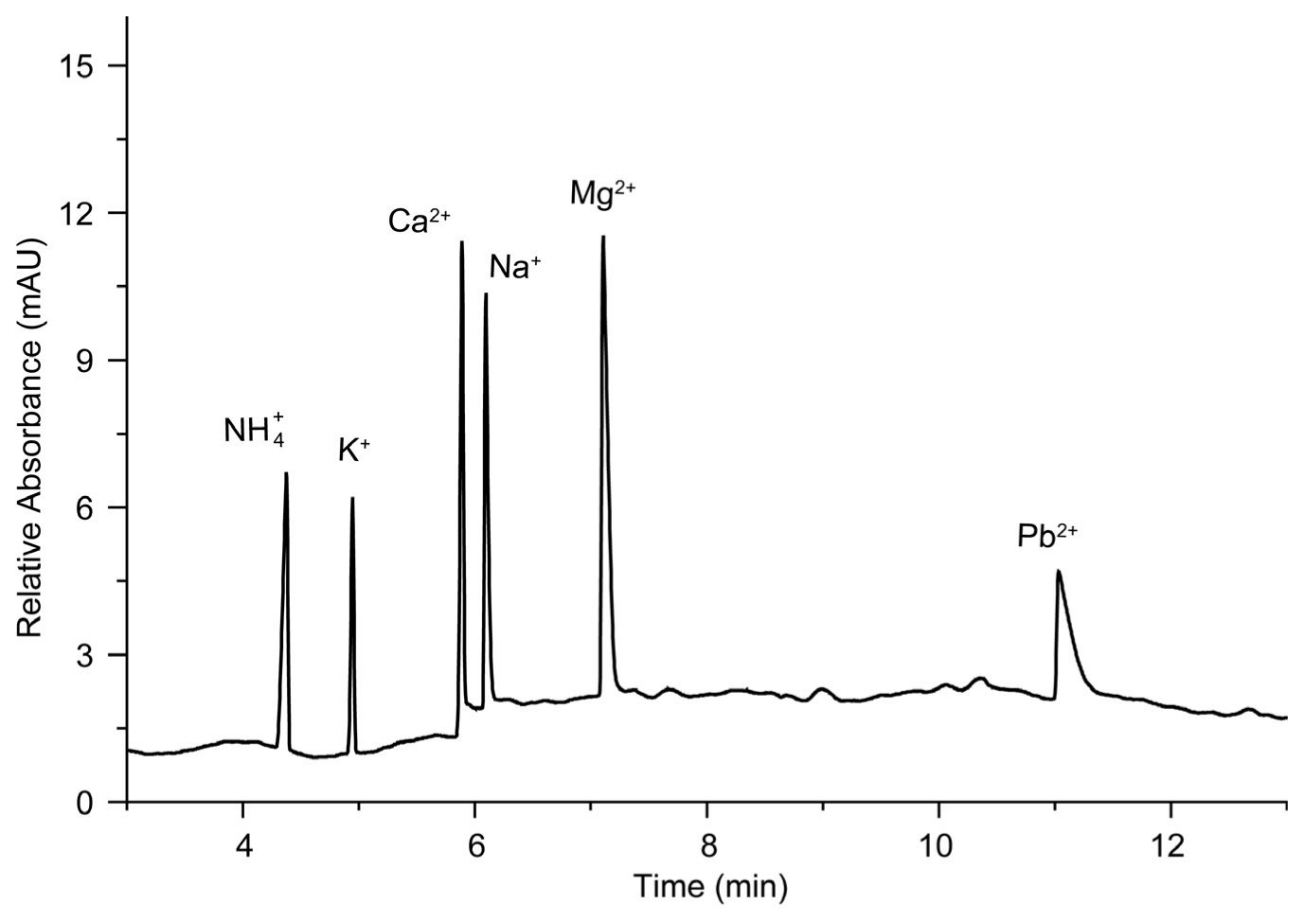
Electropherogram of reference standard cation mixture. The concentration of the ammonium, potassium, calcium, sodium, and magnesium cations was 25 ppm each, and that of lead 250 ppm. The BGE is comprised of 15 mM imidazole, 8 mM malonic acid, 2 mM 18-crown-6 ether, 10% v/v MeOH (pH 4.35 using 50% v/v acetic acid). Separation conditions: 25°C, 20 kV, electromigration injection at 5 kV for 5 s, UV signal detection at 280 nm with indirect reference of 214 nm.

Reference standards of six selected cations were prepared by the overall dilution of Agilent’s cation standard mixture (ammonium, calcium, sodium, potassium, and magnesium) to 25 ppm of each cation and the incorporation of 250 ppm lead from a prepared lead chloride stock solution. Figure 3 shows the electro-pherogram of the six cations which separated and eluted in less than 12 min in the following order: ammonium, potassium, calcium, sodium, magnesium, and lead. We note that the lead standard did not elute at lower concentrations suggesting the insensitivity of this method to lead cations. Additionally, the non-symmetrical peak shape of lead is due to the electrostacking condition [18] [19], which requires that the sample zones and the BGE to have comparable ionic strength. When ions have dissimilar, slower mobilities than the carrier electrolyte, a non-symmetrical peak shape can appear [20]. Peak shape ameliorates as the mobility of the analyte more closely matches that of the compound used for indirect detection [21]. The effects of electrolyte additives, running buffer, pH and other CE conditions on the analysis and separation of cations in drinking water and other solutions have been investigated and optimized in several recent studies [22] [23] [24] [25].

**Figure 4:**
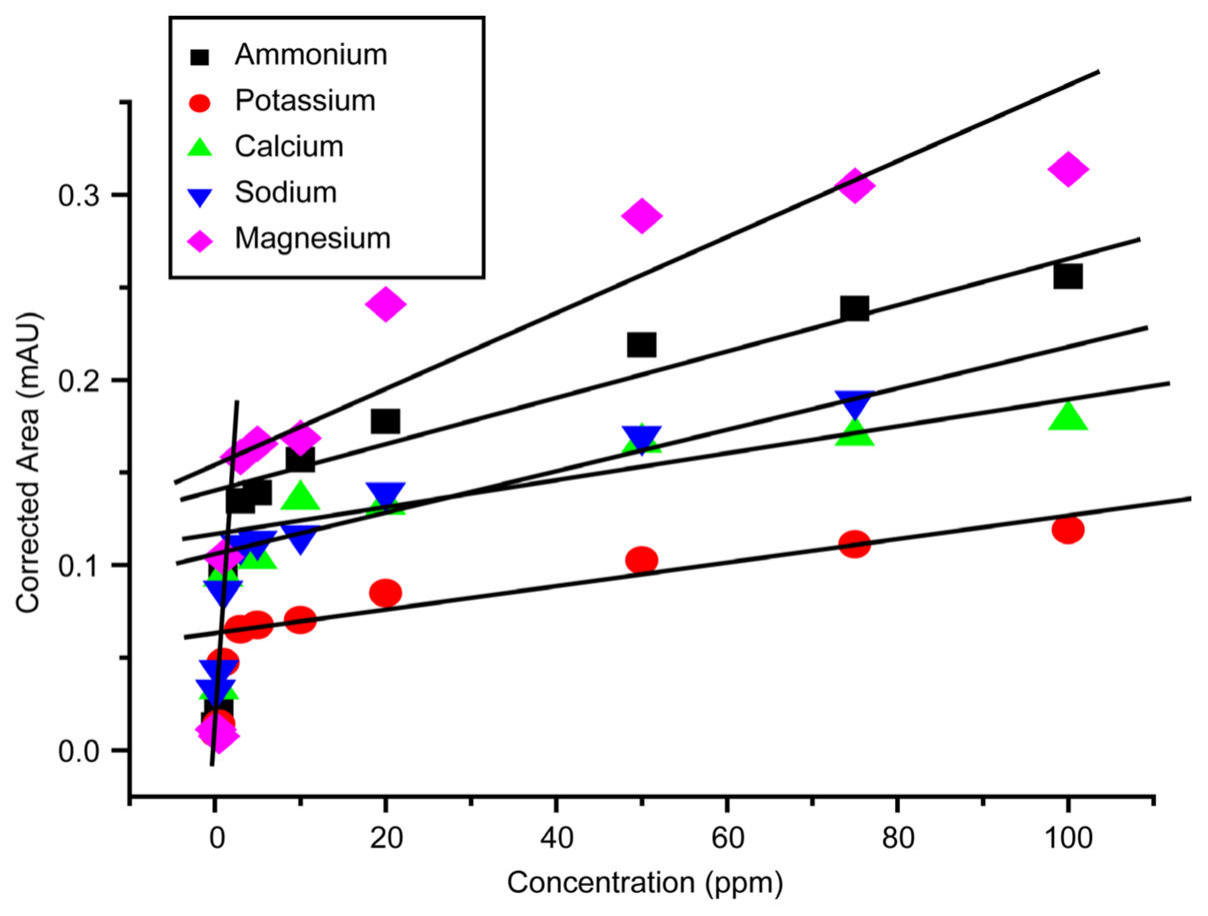
Calibration curves for five cation standards. The low cations concentration (0.1, 0.5, 1.0 and 3.0 ppm) had an average correlation of 0.88497. The linear fits for the high cations concentrations (5, 10, 20, 50, 75, and 100 ppm for ammonium, calcium, magnesium, potassium, and sodium) are shown on the figure.

To quantify the amount of detected cations in the water samples, a series of cation standards calibration curves were created (Figure 4). The concentration of the standard cation solutions of ammonium, calcium, magnesium, potassium, and sodium varied between 0.1 and 100 ppm. A break in the calibration curve’s linearity was consistently observed at around 3 ppm across all tested cations. Therefore, two calibration curves were created for each cation detected in water samples, one at low concentrations (0.1 – 3.0 ppm) and one for high concentrations (5.0 – 100.0 ppm). The goodness of the fit is determined from the coefficients of determination, R-squared (R^2^), which averaged 0.88497 for the low concentrations and 0.94819 for the high concentration calibration curves.

### 3.3. Analysis of Natural and Wastewater Sources

**Figure 5:**
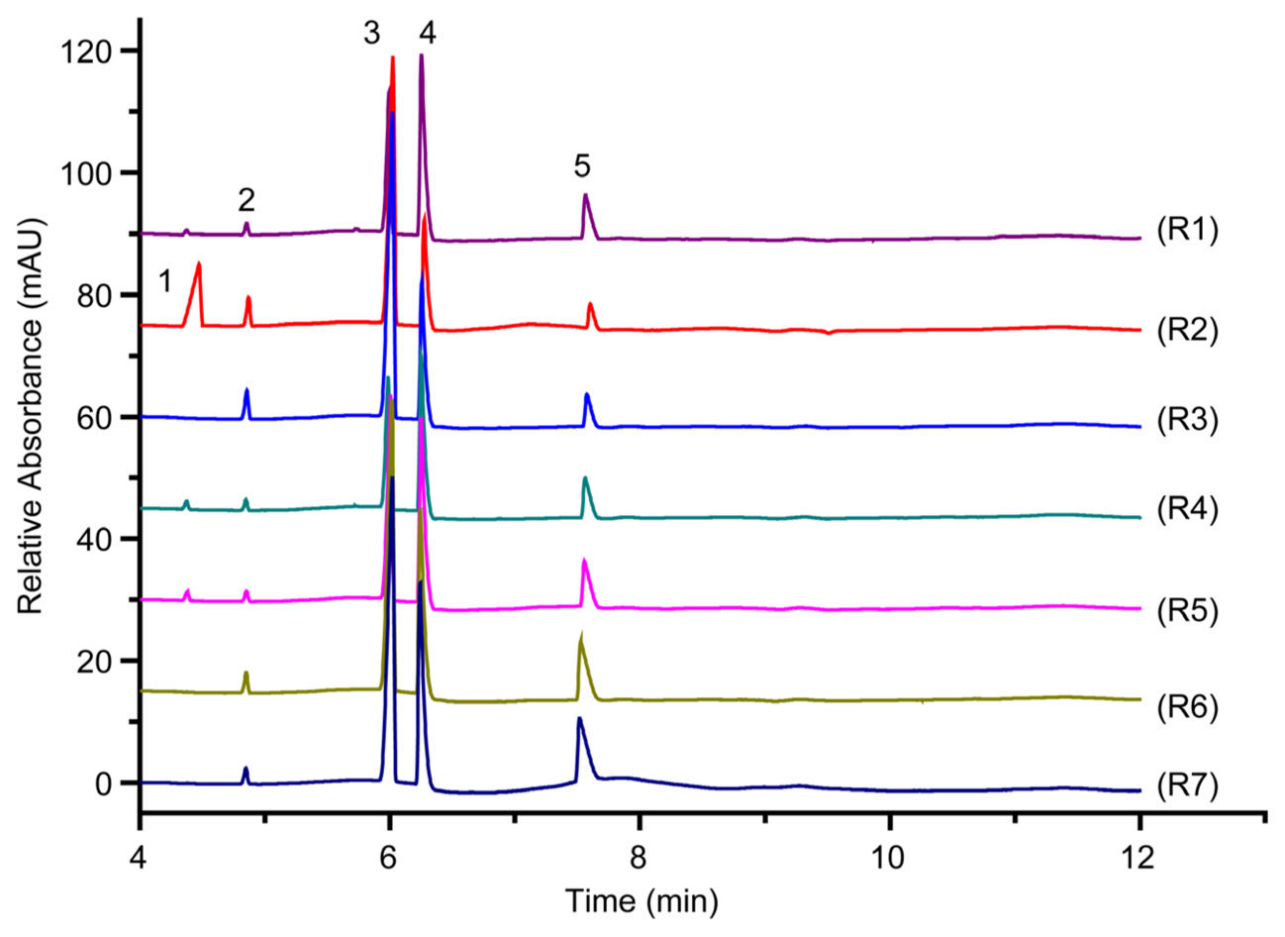
Electropherograms of water samples taken from (R1) Raquette River at the Crusher, (R2) Pre-WWT in Tupper Lake, (R3) Post-WWT in Tupper Lake, (R4) Carry Falls Reservoir, (R5) South Colton Reservoir, (R6) Pre-WWT in Potsdam, and (R7) Post-WWT in Potsdam. Conditions are as in Figure 3. Peaks: 1, ammonium; 2, potassium; 3, calcium; 4, sodium; 5, magnesium.

Natural water samples were obtained along the Raquette River watershed route, beginning at Bog River Falls, the site nearest upstream to the source (Blue Mountain Lake), continuing downstream towards the St. Lawrence River as far north as Potsdam, NY. The seven sites for which the electropherograms in Figure 5 are displayed (R1 – R7) represent the watershed and wastewater sites analyzed in this study. All tested samples contained calcium, magnesium and sodium. Lead was not detected in any of the samples. The cations detected in all the natural water samples from the Raquette River sites and wastewater sites (R1 – R7, Figure 5) were calcium, magnesium, potassium and sodium. Table 2 displays the sites, sample locations and concentrations in ppm of all the cations tested in this study. The concentration of calcium detected in the samples varied between 47 ppm and 364 ppm at the Crusher on Raquette River, upriver from Tupper Lake (R1) and Post-WWT in Tupper Lake (R3), respectively. Magnesium concentrations were found at 0.25 ppm at the Pre-WWT in Tupper Lake (R2) and 1.8 ppm at the Post-WWT in Potsdam (R7). These values are well below NYSDEC’s regulated standard concentrations of 35 ppm (Table 1). The levels of potassium ranged from trace amounts of <0.03 ppm from the Raquette River at the Crusher upriver from Tupper Lake (R1), Carry Falls Reservoir (R4), and South Colton Reservoir (R5) to 0.7 ppm at Pre-WWT in Tupper Lake (R2). Sodium concentration levels were quantified from as low as 2.5 ppm at the Pre- WWT in Tupper Lake (R2) to as high as 154 ppm at the Post-WWT in Potsdam (R7). All locations (Figure 5, R1, R3 – R7; Table 2), barring the Pre-WWT in Tupper Lake (R2), had sodium levels above NYSDEC standards of 20 ppm (Table 1), keeping in mind NYSDEC does not have regulations for wastewaters in this respect. Ammonium was detected in the samples from the Raquette River at the Crusher (R1), Carry Falls Reservoir (R4), and South Colton Reservoir (R5) in very small concentrations (<0.5 ppm), where the sample from Pre-WWT in Tupper Lake (R2) presented a concentration of 11 ppm, which is an expected detection level for wastewater. It is interesting to note that no ammonium was detected in the Pre-WWT sample from Potsdam (R6). This lack of ammonium detection in Potsdam’s wastewater could be the result of increased dilution in the sewer system causing the concentration level to drop below detection levels for our method. No ammonium was detected in either of the Post-WWT samples from Tupper Lake (R3) and Potsdam (R7).

### 3.4. Potable Water Analysis

**Figure 6:**
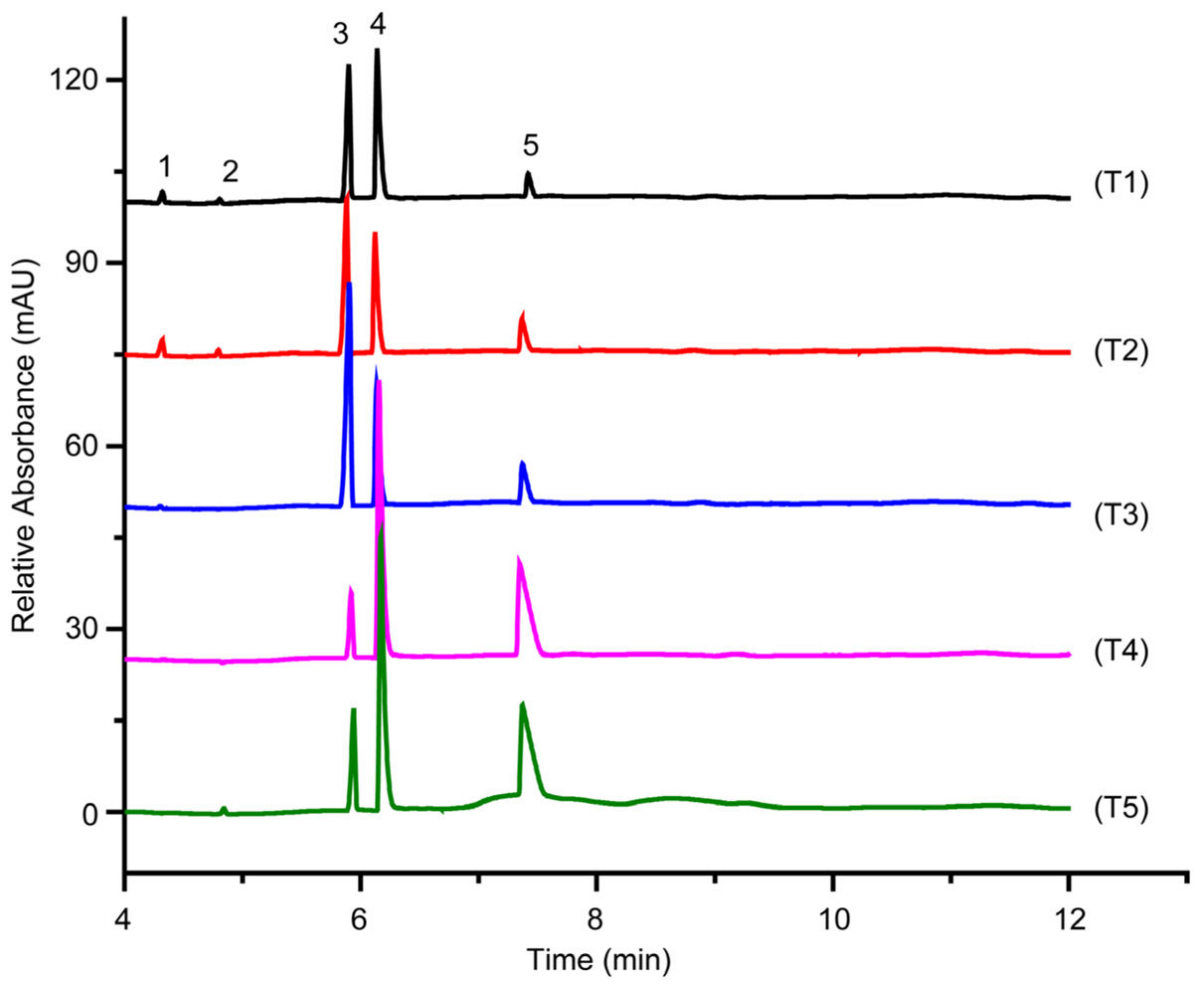
Electropherograms of tap water samples from (T1) Co-op located in village of Tupper Lake, (T2) a home located on Mt. Morris in Tupper Lake, (T3) State University of New York (SUNY) at Potsdam, (T4) St. Lawrence University (SLU) in Canton, and (T5) home in village of Canton. Conditions as in Figure 3. Peaks: 1, ammonium; 2, potassium; 3, calcium; 4, sodium; 5, magnesium.

**Figure 7:**
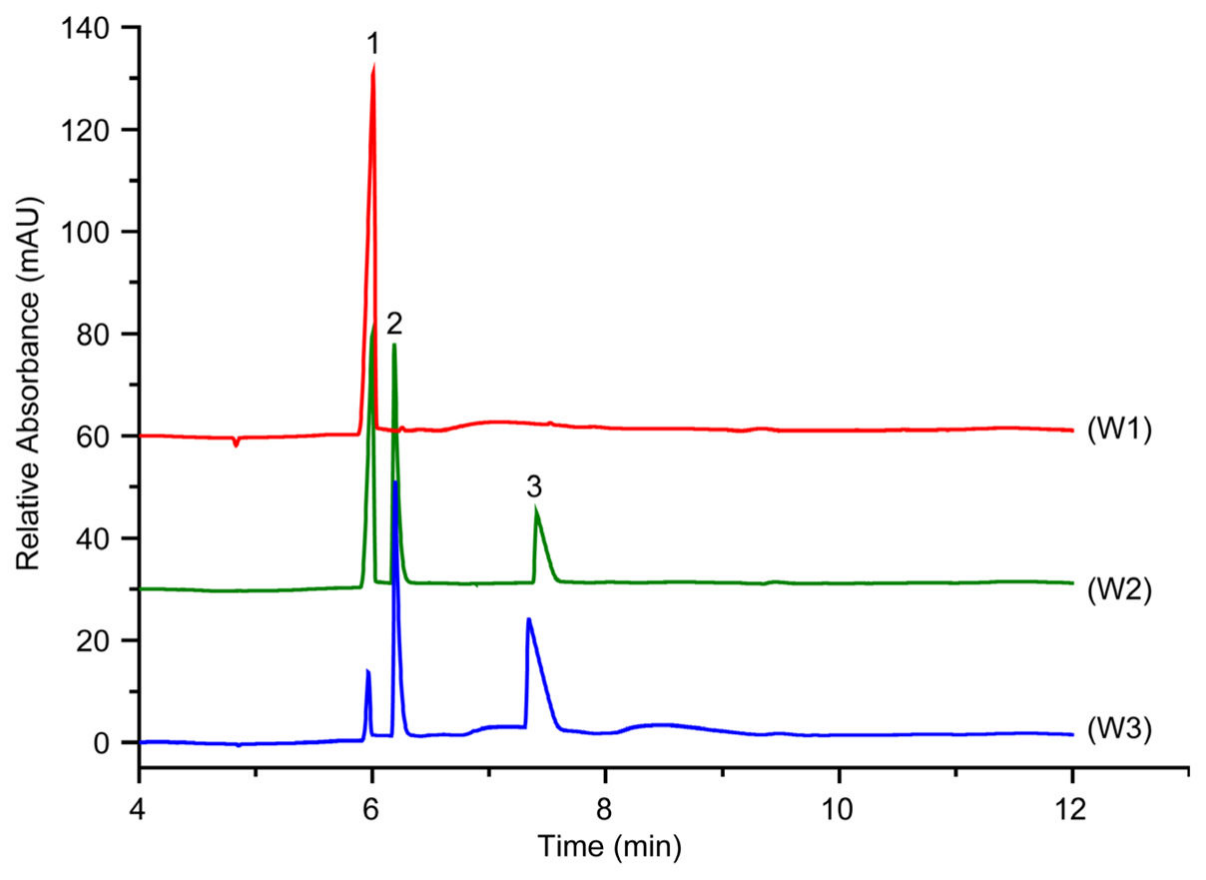
Electropherograms of well water samples from (W1) home in town of Potsdam, (W2) home in town of Canton, and (W3) home in village of Brasher Falls-Winthrop. Conditions as in Figure 3. Peaks: 1, calcium; 2, sodium; 3, magnesium.

Potable water samples were obtained from taps and wells of various homes and institutions in and around Canton, Potsdam, Tupper Lake, and Brasher Falls-Winthrop, NY. All tap and well water samples contained calcium, sodium and magnesium (T1 – T5, Figure 6 and W1 – W3, Figure 7, respectively). Calcium levels ranged from 0.83 ppm at St. Lawrence University (SLU) in Canton (T4) to 186 ppm at State University of New York (SUNY) at Potsdam (T3). Trace amounts of ammonium (~0.05 ppm) were detected in tap samples obtained from the Co-op located in the Village of Tupper Lake (T1), a home located on Mt. Morris in Tupper Lake (T2), and from this author’s laboratory at SUNY Potsdam (T3). Trace amounts of potassium (<0.03 ppm) were quantified in the samples taken from the Co-op located in the village of Tupper Lake (T1), a home located on Mt. Morris in Tupper Lake (T2), and a home in the village of Canton (T5).

None of the well water samples contained detectable levels of ammonium, lead or potassium. The well water sample obtained from a home in the town of Potsdam consisted of trace amounts of sodium (<0.05 ppm) and the highest concentration of calcium (755 ppm) than any other site tested. This concentrated level could be due to the fact that the Northern New York geologic region is known to produce hard water for those who have wells as their potable water source without a properly functional water softener. The other well water samples obtained from the homes in the town of Canton (Figure 7, W2) and village of Brasher-Falls/Winthrop (W3) had relatively high concentrations of sodium, and calcium or magnesium. This is not remarkably unusual since calcium and magnesium, in combination with sulfate and carbonate, cause water hardness and is typically found in natural and well waters, which can remain present even with salt additive in water softener systems. At least one of the detected constituents in the well water samples far exceeded the standards dictated by NYSDEC (Table 1), which constitutes only aesthetic properties in terms of the level of water hardness in the case of calcium and magnesium, and in taste for sodium. Although no lead was detected in any of the water samples tested, a more systematic study involving hundreds of samples from older homes is needed to rule out the presence of this heavy metal in the water supply.

## 4. Conclusion

Our study demonstrated that capillary zone electrophoresis (CZE) with indirect UV detection is a sensitive, reliable, and suitable method for the determination of several inorganic cations. The quick, simple and inexpensive sample preparation method makes CZE an appealing tool for the detection and quantification of many cations present in wastewaters, natural waters, and potable waters including tap and well waters. Under our experimental conditions, all tested cations eluted in under 12 min with excellent linearity and reproducibility. No lead was detected in any of the tested water samples, including wastewaters, although a more systematic study is required to rule out the presence of this toxic heavy metal in the water supply. Ammonium levels detected were well under the standard limits as dictated by NYSDEC. Although the concentrations of magnesium and sodium in several of the samples tested were above NYSDEC standards, the violation was mostly in regards to aesthetics and not adverse health effects, with the exception of excessively high sodium levels which may contribute to an increase in blood pressure.

## Figures and Tables

**Table 1:** Water-quality criteria for several cations tested in this study. All regulated standards are from US Environmental Protection Agency, US EPA [15], and New York State Department of Environmental Conservation, NYSDEC [16].

Constituent	Standard	Source or Cause	Significance
Ammonium/Ammonia ( ${\text{NH}}_{3}+{\text{NH}}_{4}^{+}$ as N)	2 mg/L**	Decomposition of animal and plant proteins, agricultural, urban runoff, and effluent from waste water treatment plants or industrial contamination.	Can cause unwanted algal blooms. Can indicate sewage pollution and consequent possible presence of pathogenic microorganisms.
Calcium(Ca^2+^)	---	Occurs in rocks, bones, shells, etc. Very abundant.	Causes water hardness. Essential for normal growth and health(1 – 2 g daily requirement).
Lead(Pb^2+^)	MCL 15 μg/L* 50 μg/L**	Leaching from ores and erosion of natural deposits; effluent discharges; corrosion of household plumbing systems.	A cumulative poison particularly in infants and children; Delays physical and mental development; May cause kidney problems and high blood pressure in adults.
Magnesium (Mg^2+^)	35 mg/L**	Major constituent of geological formations.	A major dietary requirement for humans (0.3 –0.5 g/day)and the second major constituent of water hardness.
Potassium (K^+^)	---	Geological formations.	No health concerns except at gross levels.
Sodium(Na^+^)	20 mg/L**	Abundant constituent of rocks and soils.	Essential dietary requirement (5 g or more ). Causes hypertension if in excess.

MCL, Maximum Contaminant Level (highest level of a contaminant that is allowed in drinking water); ---, no limit established;

* US EPA standard;

** NYSDEC standard.

**Table 2:** Concentration of inorganic cations (ppm) detected in water samples tested in this study. Each sample was run at least three times to ensure reproducibility and the results below are average values from multiple experiments with less than 5% uncertainty. The high ppm values of some of the cations were estimated by extrapolation using the calibration curves of Figure 4.

		Concentration levels (ppm)					
Site	Sample location	${\text{NH}}_{4}^{+}$	K^+^	Ca^2+^	Na^+^	Mg^2+^	Pb^2+^
R1	Raquette River, upriver from Tupper Lake, at the Crusher	<0.05	<0.03	47	85	0.79	---
R2	Pre-WWT in Tupper Lake	11	0.7	254	2.5	0.25	---
R3	Post-WWT in Tupper Lake	---	0.62	364	47	0.52	---
R4	Carry Falls Reservoir	<0.05	<0.03	52	80	0.85	---
R5	South Colton Reservoir	<0.05	<0.03	144	122	0.97	---
R6	Pre-WWT in Potsdam	---	0.034	301	112	1.5	---
R7	Post-WWT in Potsdam	---	<0.03	357	154	1.8	---
T1	Tap water, village of Tupper Lake	<0.05	<0.03	50	63	0.21	---
T2	Tap water, Mt. Morris area in Tupper Lake	0.054	<0.03	73	19	0.51	---
T3	Tap water, SUNY Potsdam	<0.05	---	186	19	0.74	---
T4	Tap water, SLU in Canton	---	---	0.83	282	29	---
T5	Tap water, village of Canton	---	<0.03	3.6	260	88	---
W1	Well water, Town of Potsdam	---	---	755	<0.5		---
W2	Well water, Town of Canton	---	---	353	281	3.1	---
W3	Well water, Winthrop	---	---	1.8	315	221	---
